# Coverage of neuroma in continuity

**DOI:** 10.1186/1753-6561-9-S3-A76

**Published:** 2015-05-19

**Authors:** Roberto Adani

**Affiliations:** 1 Department of Hand Surgery, Azienda Ospedaliera Universitaria Integrata Verona, Verona, 37134, Italy

## 

Neurogenic pain can develop after microsurgical repair of a median nerve injury or when the lesion is underestimated and therefore not treated at all. Nerve injury with neuromatous pain may be associated with tumefaction at the wrist, increased pain and tingling when tapped [1]. The aim of the treatment is to both minimize pain and preserve residual function of the median nerve.

Surgical procedures such as neurolysis do not always relieve the patients’ pain. Reconstruction with grafts is reserved only for cases with very poor sensory and motor recovery. Neurolysis should be performed carefully to avoid devascularization of the nerve and formation of a new scar around the nerve [2]. Particular attention has been given to the coverage and wrapping of a neuroma with different pedicle flaps as the pronator quadratus muscle flap [3], the Becker flap [4], the reverse island radial fascial flap with or without inclusion of the radial artery [2,5-7] and the local synovial flap [8]. With regards to free flaps, Wintsch [9] believes that a thin flap of gliding fascia is the ideal flap to cover a nerve, whereas Jones [10] advocates the use of a small hemi-latissimus dorsi muscle flap. The lateral arm flap [2,11,12] and the scapular flap [2,11] represent other options but the thickness of the subcutaneous tissue and the overlying skin often produce very prominent flaps [10]. Goitz and Steichen [13] reported the coverage of a scarred median nerve with a free omental flap.

The purpose of this study is to review the treatment of painful neuroma of the median nerve at the wrist treated with external neurolysis and coverage using the ulnar artery perforator adipofascial flap (UAPAF) or the radial artery perforator adipofascial flap (RAPAF).

## Surgical technique

External neurolysis of the median nerve was performed in every patient. Neurolysis was done under microscopic magnification to avoid nerve damage.

### Ulnar artery perforator adipofascial flap

This flap is supplied by the dorsal branch of the ulnar artery. This artery is located on a line drawn between the pisiform bone and the medial epicondyle and originates 4 cm proximally to the pisiform bone [4]. The location of the dorsal branch of the ulnar artery is usually confirmed by Doppler sonography [14]. This is a constant artery with a diameter of 1 to 1.3 mm passing beneath the flexor carpi ulnaris (FCU) tendon [4,15-17]. The artery runs in an oblique line from the volar to the dorsoulnar surface of the distal forearm [15]. The artery divides into descending and ascending branches; the latter is responsible for the vascularization of the UAPAF [18,19] which measures up to 5-9 cm in width and 10-20 cm in length [15,16,18,19].

An incision is performed around the wrist crease in an ulnar direction to allow neurolysis of the median nerve and it is then continued proximally along the mid-lateral ulnar border of the forearm for 10-12 cm. The adipofascial flap is centred over the ulnar artery on the line joining the pisiform and the medial epicondyle. Thin skin flaps are carefully elevated to allow some fat to remain on the undersurface of the dermis. The subcutaneous tissue is exposed leaving part of the superficial venous plexus with the adipofascial flap. Flap elevation is begun by incising the subcutaneous tissue through the deep fascia along the radial border of the exposure, avoiding injury to the median nerve and its palmar cutaneous branch. The ulnar artery is exposed by gentle retraction of the flap. The artery is dissected away from the flap along its course proximal to the location of the dorsal branch of the ulnar artery. When the flap reaches a suitable length (10-12 cm from the wrist) it is cut proximally and along the ulnar side; usually the width of the flap is from 4 to 6 cm. The flap is dissected proximally to distally in continuity with the underlying fascia. When the dissected flap is 4 cm proximal to the wrist joint dissection is interrupted to verify the presence and the precise location of the dorsal branch of the ulnar artery and its associated veins. Ulnar retraction of the FCU permits identification of the perforator. While identifying the dorsal branch of the ulnar artery, attention is paid to the dorsal cutaneous branch of the ulnar nerve that may be encountered during this phase [15]. Usually the pivot point of the flap is localized 4 cm proximal to the pisiform bone [16]. The adipofascial flap is turned over by 180° and finally anchored with a few absorbable stitches around the median nerve and the skin is loosely sutured.

### Radial artery perforator adipofascial flap

The flap is supplied by the septocutaneous perforators of the distal radial artery [5,20,21]. The radial artery at the distal forearm emerges in the septum between the flexor carpi radialis (FCR) and the brachioradialis tendons to give about 10 small perforating vessels with an external diameter between 0.3 and 0.9 mm [20-24]. These vessels arise approximately 1.5 cm proximally to the radial styloid and recur proximally at intervals of 0.4 to 1.5 cm [21]. A very rich venous plexus is accompanying this arterial network. For most authors the safest pivot point of the pedicle is about 2 to 4 cm above the radial styloid process [20,23,24]. Doppler examination would be useful to identify the radial artery perforators, but it may not be necessary given their reliability [24].

The proximal margin of the flap is located 10 cm distal to the elbow and the base of the flap is 2 to 4 cm proximal to the radial styloid process [22,23]. Flap dimensions reported in literature range from 8-12 cm in width and 15-20 cm in length [23,24].

An incision is made on the wrist crease and after neurolysis of the median nerve, the incision is prolonged proximally for 10-14 cm along the axis of the radial artery. The skin is then carefully elevated in order to maintain some adipose tissue with the skin. In this phase the dissection is very similar for the UAPAF. The incision is deepened down to the deep fascia at the margins of the flap radially over the brachioradialis muscle and ulnarly over the FCR muscle with the width of the flap ranging from 5 to 6 cm. The proximal margin of the flap is incised 10 to 12 cm proximal to the wrist. The adipofascial flap is elevated together with the deep fascia away from the radial artery leaving it intact. Dissection is carried on distally until the pivot point is reached, about 4 cm proximal to the radial styloid with a flap base narrowed to about 3 to 4 cm in width. The lateral forearm nerves, including the sensory superficial radial nerve and the lateral antebrachial cutaneous nerve, should be identified and carefully preserved so as to avoid injury and potential subsequent painful neuroma formation. In this procedure there is no need to identify the perforator vessels around the radial styloid and they should not be dissected in order to avoid damage. The flap is turned over by 180° to cover the median nerve and the tourniquet is then released to visualize the blood supply of the flap.

The technique used in this study included subcutaneous fat taken with the fascial flap. This did not compromise vascularization of the skin of the forearm; moreover skin closure was done without excessive tension. This bulkier flap can cover the median nerve superficially with a padding tissue composed by the fascia and the adipose tissue wrapping the nerve deeply with the fascia only. In this way the median nerve is completely isolated from the adjacent finger flexor tendons and protected against external trauma. Pedicle perforator adipofascial flaps represent a safe and reliable procedure: they are harvested easily and quickly with an adequate blood supply with loupe magnification. In this study it was not possible to observe significant differences in terms of surgical technique or functional results between the two flaps used. It is therefore difficult to give definitive conclusions about the best flap to use: both can be done under axillary plexus block and the final decision depends on the local conditions and the surgeon’s preference. We find the RAPAF procedure easier because the perforators do not require dissection. Also the RAPAF allows raising a longer, wider and thicker flap than the UAPAF.

Five out of the eight patients had complete resolution of pain after this procedure while two patients had partial resolution. This study is limited by the small number of patients. A prospective study is needed to ascertain the potential superiority of nerve coverage over other currently employed techniques. Also a larger number of patients collected from different centers using the same surgical procedures would support the validity of this approach.
